# Oxidative Stress Is Not a Major Contributor to Somatic Mitochondrial DNA Mutations

**DOI:** 10.1371/journal.pgen.1003974

**Published:** 2014-02-06

**Authors:** Leslie S. Itsara, Scott R. Kennedy, Edward J. Fox, Selina Yu, Joshua J. Hewitt, Monica Sanchez-Contreras, Fernando Cardozo-Pelaez, Leo J. Pallanck

**Affiliations:** 1 Department of Genome Sciences, University of Washington, Seattle, Washington, United States of America; 2 Molecular and Cellular Biology Program, University of Washington, Seattle, Washington, United States of America; 3 Department of Pathology, University of Washington, Seattle, Washington, United States of America; 4 Postbaccalaureate Research Education Program, University of Washington, Seattle, Washington, United States of America; 5 Department of Neuroscience, Mayo Clinic, Jacksonville, Florida, United States of America; 6 Department of Biomedical and Pharmaceutical Sciences, University of Montana, Missoula, Montana, United States of America; Newcastle University, United Kingdom

## Abstract

The accumulation of somatic mitochondrial DNA (mtDNA) mutations is implicated in aging and common diseases of the elderly, including cancer and neurodegenerative disease. However, the mechanisms that influence the frequency of somatic mtDNA mutations are poorly understood. To develop a simple invertebrate model system to address this matter, we used the Random Mutation Capture (RMC) assay to characterize the age-dependent frequency and distribution of mtDNA mutations in the fruit fly *Drosophila melanogaster*. Because oxidative stress is a major suspect in the age-dependent accumulation of somatic mtDNA mutations, we also used the RMC assay to explore the influence of oxidative stress on the somatic mtDNA mutation frequency. We found that many of the features associated with mtDNA mutations in vertebrates are conserved in *Drosophila*, including a comparable somatic mtDNA mutation frequency (∼10^−5^), an increased frequency of mtDNA mutations with age, and a prevalence of transition mutations. Only a small fraction of the mtDNA mutations detected in young or old animals were G∶C to T∶A transversions, a signature of oxidative damage, and loss-of-function mutations in the mitochondrial superoxide dismutase, *Sod2*, had no detectable influence on the somatic mtDNA mutation frequency. Moreover, a loss-of-function mutation in *Ogg1*, which encodes a DNA repair enzyme that removes oxidatively damaged deoxyguanosine residues (8-hydroxy-2′-deoxyguanosine), did not significantly influence the somatic mtDNA mutation frequency of *Sod2* mutants. Together, these findings indicate that oxidative stress is not a major cause of somatic mtDNA mutations. Our data instead suggests that somatic mtDNA mutations arise primarily from errors that occur during mtDNA replication. Further studies using *Drosophila* should aid in the identification of factors that influence the frequency of somatic mtDNA mutations.

## Author Summary

Mitochondria are the evolutionary remnants of bacteria that were acquired by the cells of our ancestors more than a billion years ago and now produce virtually all of the cellular energy. Due to their bacterial ancestry, mitochondria have their own genomes, which encode some of the machinery responsible for producing energy. These genes occasionally acquire mutations—irreversible alterations that can adversely affect the energy production machinery. The accumulation of mitochondrial DNA (mtDNA) mutations is thought to cause aging and common age-related diseases, but we know little about the factors that influence the frequency of these mutations. Our study tested whether fruit flies would serve as a good animal model to study this problem. We found that flies accumulate mtDNA mutations in a pattern similar to that of humans. We then used flies to test the long-standing theory that toxic free radicals, chemical byproducts of energy production, cause mtDNA mutations to accumulate. Our data do not support this hypothesis, and instead suggest that rare errors associated with duplicating mitochondrial genomes are primarily responsible for mtDNA mutations. In sum we demonstrate that *Drosophila* serves as a tractable genetic model to investigate the mechanisms that influence the frequency of somatic mtDNA mutations.

## Introduction

Mitochondria play crucial cellular roles in energy production, Ca^2+^ buffering, metabolite synthesis, and programmed cell death in metazoans [1]–[3]. While most mitochondrial proteins are encoded in the nuclear genome, mitochondria also contain a compact genome that generally encodes 37 genes [4], and germline mutations that disrupt the functions of mitochondrial DNA (mtDNA) encoded genes cause a number of devastating familial syndromes [5]. mtDNA mutations also occur in somatic tissues, and the accumulation of somatic mtDNA mutations is implicated in aging and common diseases of the elderly, including cancer, diabetes, and neurodegenerative disease [5]–[8]. Because there are multiple copies of mtDNA in any given cell, when mtDNA mutations occur, they frequently coexist with wild-type (WT) mtDNA, a condition known as heteroplasmy. For reasons that are not presently understood, many mtDNA mutations expand clonally within a cell, such that a single somatic mtDNA mutation can ultimately represent a large fraction of the mtDNA within a given cell or tissue [9]. Although the ratio of mutated to WT mtDNA is believed to play a critical pathological role in diseases associated with mtDNA mutations [10], the molecular mechanisms that influence this ratio are poorly understood.

The frequency of somatic mtDNA mutations can exceed the mutation frequency of the nuclear genome by several orders of magnitude [10], and various factors have been proposed to account for this high mutation frequency. Because mitochondria are the major cellular source of DNA-damaging reactive oxygen species (ROS), it is believed that ROS-mediated damage is an important contributor to somatic mtDNA mutations. Indeed, Harman proposed in the 1970s that ROS damage to mtDNA causes mtDNA mutations that result in production of dysfunctional respiratory chain components, which in turn produce increased amounts of ROS, thus leading to a vicious cycle responsible for aging [11]. Another possible source of the high mtDNA mutation frequency is mtDNA replication errors. Unlike the nuclear genome, which does not replicate in postmitotic tissues, mtDNA replicates throughout life in postmitotic tissues [10], and this ongoing replication can potentially lead to the accumulation of replication errors. Finally, the relative lack of particular DNA repair pathways and protective histones in mitochondria has also been suggested as a source of the high frequency of mtDNA mutations [12]. However, the relative contributions of these and other possible causes of the age-dependent accumulation of somatic mtDNA mutations remain unclear.

Recent work in *Drosophila melanogaster* has contributed greatly to our understanding of mitochondrial quality control [13], [14], so we sought to use *Drosophila* as a simple, genetically tractable model system to explore the factors that influence somatic mtDNA mutations. Although much is known about the frequency and distribution of somatic mtDNA mutations in vertebrates [10], these matters are largely unexplored in invertebrates. Only three previous studies have attempted to test whether somatic mtDNA mutations accumulate in *Drosophila*, and these studies have yielded contradictory findings [15]–[17]. While technical issues likely explain these discordant findings, a limitation of all three studies is the lack of quantitative estimates of the somatic mtDNA mutation frequency, including the somatic mtDNA point mutation frequency. Thus, we used an approach that would enable us to address these issues in *Drosophila*.

To explore the distribution, frequency, and causes of somatic mtDNA mutations in *Drosophila*, we used the Random Mutation Capture (RMC) assay [18], [19]. We found that the somatic mtDNA mutation frequencies in fly tissues are similar to those reported in vertebrates, and that their frequencies increase with age. However, the specific types of mtDNA mutation detected, and the results of gene perturbations targeting oxidative stress and repair pathways, indicate that oxidative stress is not a major contributor to the somatic mtDNA mutation frequency. Instead, our findings suggest that somatic mtDNA mutations arise primarily from errors that occur during DNA replication.

## Results

### mtDNA replication occurs in somatic tissues of *Drosophila*

Previous work has shown that mtDNA synthesis occurs in mitochondria isolated from adult flies [20] and that mtDNA replication intermediates can be detected in adult *Drosophila* tissues [21], suggesting that mtDNA replication occurs in the somatic tissues of adult *Drosophila*. Given that DNA replication is essential to the formation of point mutations we performed an additional experiment to verify that mtDNA replication occurs in somatic tissues of *Drosophila* adults. We fed adult flies 5-bromo-2′-deoxyuridine (BrdU) and used Southwestern blot analysis and immunofluorescence confocal microscopy to test whether this thymidine analog was incorporated into mtDNA. After 72 hours of exposure to BrdU, DNA was isolated from *Drosophila* heads, digested with BglII, then subjected to Southwestern blot analysis using antiserum against BrdU. Because the *Drosophila* mitochondrial genome is 19.5 kb in size and contains only one BglII site, BrdU labeling should yield a single 19.5 kb band. Consistent with this prediction, our analysis revealed a single 19.5 kb band, indicating BrdU incorporation into mtDNA (**Figure S1A**). To further validate this finding, we used immunohistochemistry and confocal microscopy to test whether BrdU immunofluorescence colocalizes with mitochondria in the *Drosophila* thoracic ganglion. After feeding adult flies BrdU for 72 hours, the thoracic ganglion was dissected and stained with MitoTracker Deep Red to detect mitochondria and then incubated with an antibody against BrdU. Confocal microscopy revealed anti-BrdU immunofluorescence that colocalized with MitoTracker Deep Red fluorescence in BrdU-labeled animals, but not in unlabeled controls (**Figure S1B**). The finding that only a subset of mitochondria stained positively for BrdU is consistent with previous work indicating that mtDNA replication occurs at a low rate in the nervous system [22]. While we cannot rule out the possibility that some mitochondrial BrdU labeling is due to repair of mtDNA, our data together with previous work supports the occurrence of mtDNA replication in the adult somatic tissues of *Drosophila* [20], [21].

### Multiple *TaqI* sites are suitable for detecting mutations in *Drosophila* mtDNA

A major challenge in detecting somatic mtDNA mutations is that they arise independently in different cells, such that any single mtDNA mutation typically lacks sufficient abundance within a tissue to be reliably detected by direct sequencing [23], [24]. Thus, we chose to use the Random Mutation Capture (RMC) assay to quantify the mtDNA mutation frequency in *Drosophila* because this assay can detect sequence variants at a detection limit of 10^−8^ [9]. The RMC assay measures the frequency at which a particular *TaqI* restriction site in mtDNA is eliminated through mutation. The RMC assay is a qPCR-based assay that uses two PCR primer sets. One primer set is used to amplify a portion of the mtDNA that contains a single *TaqI* restriction site (the test primers); the other primer set is used to amplify a portion of mtDNA that lacks a *TaqI* restriction site (the control primers). These primer sets are then used in qPCR reactions with *TaqI*-digested mtDNA to estimate the total number of mtDNA molecules present in the sample (determined by the qPCR reaction containing the control primer set) and the number of mtDNA molecules bearing mutations that eliminate the *TaqI* site (determined by the qPCR reaction containing the test primer set). In addition to the exquisite sensitivity with which RMC can detect mtDNA mutations, another advantage of this method is that WT molecules are eliminated by *TaqI* digestion prior to PCR amplification, and thus PCR amplification errors do not confound the mtDNA mutation frequency estimate. Previous work indicates that the majority of mtDNA mutations detected with this method are single base pair substitutions; however, small deletions and insertions can also be detected [25].

**Figure 1:**
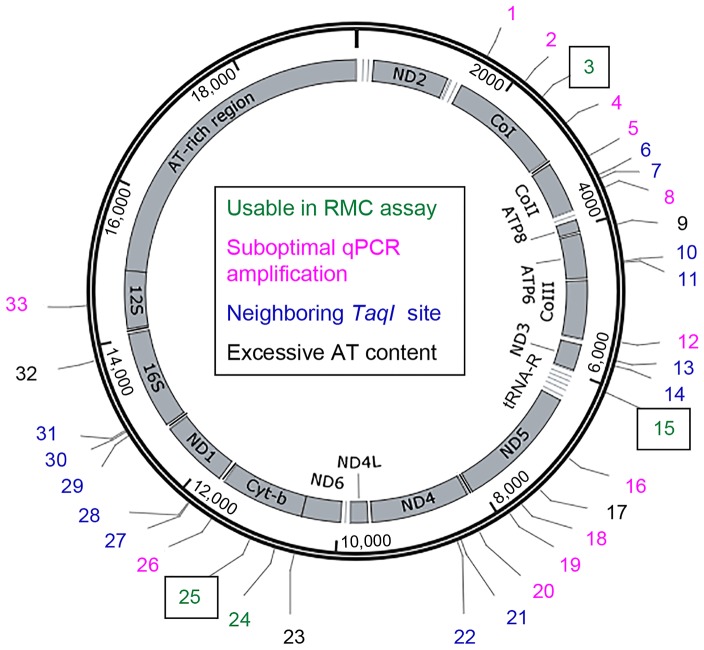
The locations of *TaqI* sites in *Drosophila* mtDNA. Schematic depiction of the *Drosophila melanogaster* mitochondrial genome. Numbers (1-33) indicate the different *TaqI* sites, color coded according to their utility for RMC analysis as indicated. Boxed numbers indicate the *TaqI* sites used for RMC analysis in our work. tRNA genes are represented by horizontal lines adjacent to other mitochondrial genes.

The *D. melanogaster* mtDNA sequence contains 33 *TaqI* sites (**Figure 1**). Because the mutability of these sites could vary, we sought to measure the mtDNA mutation frequency at multiple *TaqI* sites (**Figure 1** **, Table S1**). Four *TaqI* sites were excluded from analysis because the extreme AT-richness of the flanking sequence precluded primer design. Another 13 *TaqI* sites were excluded because their proximity to one or more adjacent *TaqI* sites did not allow for the design of primer sets that amplify only a single *TaqI* site. We designed primer pairs to the flanking sequences of the remaining 16 *TaqI* sites and used them in experiments aimed at qPCR primer optimization (see Materials and Methods). Only 4 out of these 16 primer sets had optimum amplification profiles (**Table S1**). These 4 primer sets amplified *TaqI* sites in three different genes, and we used three of these test primer sets (corresponding to three different genes) for our studies. The three *TaqI* sites interrogated in our study are located in *mt:Cyt-b* (Complex III subunit), *mt:CoI* (Complex IV subunit), and *mt:tRNA:Arg* (**Figure 1** **, Table S1**). The control primer pair anneals to sequences in the *mt:CoIII* gene (Complex IV subunit) (**Table S1**).

### Somatic mtDNA mutations accumulate with age in *Drosophila*

To explore the frequency and distribution of somatic mtDNA mutations in *Drosophila*, we used an isogenic fly line (*w^1118^*) to control for the possible influence of nuclear genetic background on the mtDNA mutation frequency in comparisons between cohorts. We isolated mtDNA separately from the heads and thoraces of adult flies, which contain primarily nervous tissue and muscle tissue, respectively so that the somatic mtDNA mutation frequency could be compared in these tissues. The abdomen was excluded from analysis because mtDNA replication in the female germline might confound our analysis of the somatic mtDNA mutation frequency.

**Figure 2:**
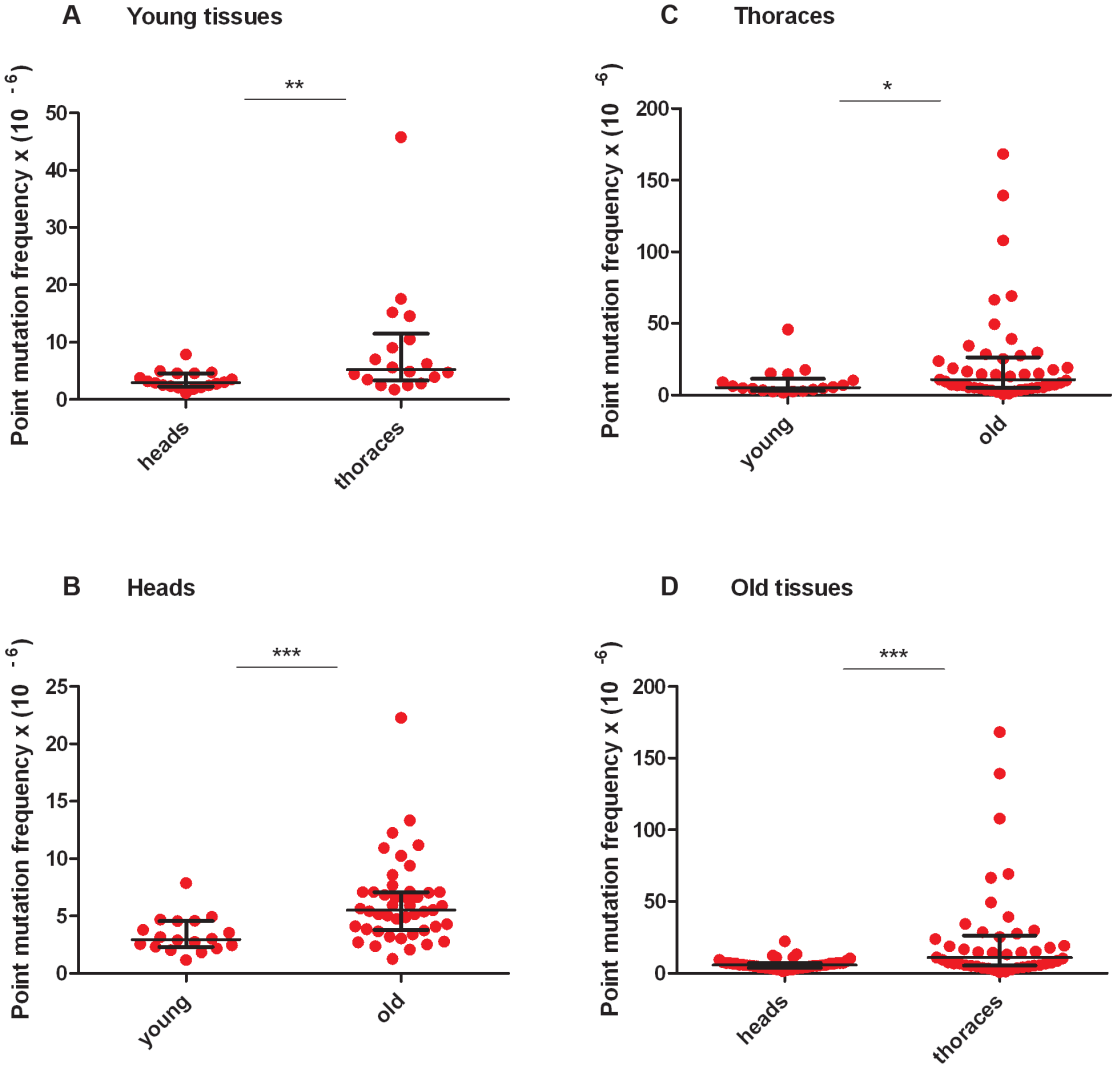
Mitochondrial DNA mutation frequency increases with age and is greater in thoraces than in heads. (**A**) The mtDNA mutation frequency is greater in young thoraces than in young heads (**p<0.01). (**B**) The mtDNA mutation frequency is greater in old heads than in young heads (***p<0.001) (**C**) and is greater in old thoraces than in young thoraces (*p<0.05). (**D**) The mtDNA mutation frequency is greater in old thoraces than in old heads (**p<0.001). Horizontal bars represent the median mutation frequency, and error bars indicate the interquartile range. Significance was tested using Mann-Whitney unpaired U tests.

RMC analysis of mtDNA isolated from the heads and thoraces of 1- to 3-day-old flies (hereafter termed “young” animals; **Figure S2B, Table S2, Table S3**) revealed a similar mtDNA mutation frequency between the three *TaqI* sites within the same tissue for either young heads or thoraces (**Figure S2**). Therefore, we pooled the data from the three *TaqI* sites to achieve greater statistical power, and detected a small but significant increase in the mtDNA mutation frequency in young thoraces relative to heads (**Figure 2A**). The mtDNA mutation frequency in heads and thoraces (∼10^−5^) was similar to the mtDNA mutation frequency reported in vertebrates [25], [26], and variation in the mtDNA mutation frequency between tissues has also been reported in vertebrates [25], [26].

We next asked whether the frequency of somatic mtDNA mutations increases with age in *Drosophila*. Prior to measuring the mtDNA mutation frequency in older flies, we performed a lifespan analysis on the *w^1118^* strain to identify a suitable age range for RMC experiments (**Figure S2A**). We used flies aged between 40 and 66 days (hereafter termed “old” animals) for our studies. As in young tissues, the mutation frequency was similar between *TaqI* sites within the same tissue for either old heads or thoraces (**Figure S2C, S2D, Table S2, Table S3**). Using pooled data from *TaqI* sites, RMC analysis revealed a significant increase in mtDNA mutation frequency with age in both heads and thoraces (**Figure 2B, C**). As in young animals, the mtDNA mutation frequency was higher in thoraces than in heads from old animals (**Figure 2D**). In contrast to previous work in mice showing an exponential increase in the frequency of mtDNA mutations with age [25], our data fit best to a model of linear increase in the mutation frequency with age (**Figure S3**). However, this discrepancy might simply reflect the dramatic increase in mtDNA mutation frequency that occurs in mice near the end of life when only 10% of the population is still alive [**25**], and the fact that the oldest flies examined in our studies involved an age where 25% of the population was still alive.

Upon closer inspection of our data, it appeared that much of the age-dependent increase in the mtDNA mutation frequency was caused by outlier samples with high mtDNA mutation frequencies (**Figure 2**). Because mtDNA mutations expand clonally in humans, we hypothesized that these outlier samples represent rare events in which a mutation within the *TaqI* site occurred early in development, and then clonally expanded in just one of the 100 flies in the sample. Assuming that a single fly fully accounts for the mtDNA mutation frequency in the outlier sample with the highest mtDNA mutation frequency, we estimate that the frequency of the clonally expanded mutation within that fly would approach 0.2%. An mtDNA mutation frequency of 0.2% is well below the threshold required to cause disease in humans for all known pathogenic mtDNA mutations [27], and thus would likely be well tolerated in flies even in the event of a deleterious mutation. A prediction of the hypothesis that outlier samples with high mtDNA mutation frequencies are a consequence of a single fly with an abundant clonally expanded mutation is that these samples should have a single predominating mutation type. By contrast, samples with an mtDNA mutation frequency nearer to the mean (non-outliers) would be more likely to contain a mixture of different mutation types that are contributed by multiple individuals in the population.

To test the hypothesis that outlier samples with high mtDNA mutation frequencies represent clonal expansion events, we compared the distribution of mutations in outlier and non-outlier samples by cloning and sequencing *TaqI* resistant DNA from these samples (**Figure S4**). We defined outlier samples as those that lie outside the 95% confidence interval of the mean for a given group. Our analysis revealed a trend towards an increased frequency of the predominating mutation in outlier samples, with the frequency greatest in the head and thorax samples with the highest mutation frequencies. However, this difference did not reach statistical significance (p = 0.09, Student's t-test). Thus, our data suggests that clonal expansion may be the explanation for the high mutation frequency in outlier samples; however, it does not allow us to rule out other explanations for outlier samples, including simple chance deviations from the mean.

### Oxidative stress is only a minor contributor to somatic mtDNA mutations

**Figure 3:**
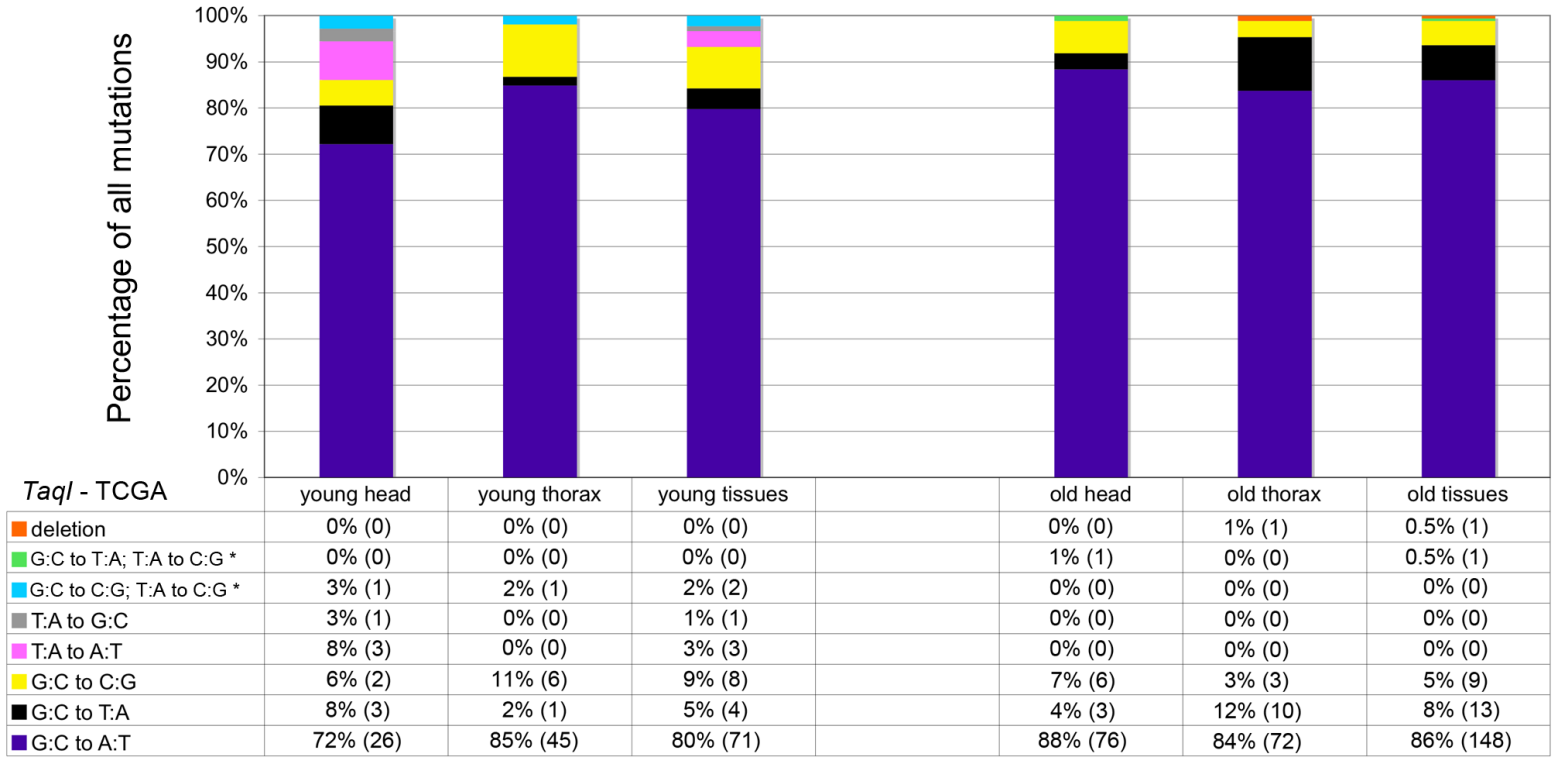
GC∶TA transversions account for only a small percentage of mtDNA mutations. The percentage and total number (in parentheses) of each type of mutation identified at *TaqI* sites in young and old animals. Results depict data obtained separately from heads and thoraces and also combined. The wild-type *TaqI* sequence (TCGA) is shown for reference. * = Two mutations identified within a single cloned *TaqI* site.

Mitochondria are major producers of superoxide anion [28], which can damage DNA [29] and is implicated in the age-dependent accumulation of somatic mtDNA mutations [11], [30], [31]. To test whether superoxide and other ROS are a major cause of somatic mtDNA mutations in *Drosophila*, we performed several experiments. First, we analyzed the mtDNA mutation spectrum using data obtained from cloning and sequencing RMC processed samples. Superoxide anion reacts with deoxyguanosine to produce 8-hydroxy-2′-deoxyguanosine (8-oxo-dG) [32], which results in G∶C to T∶A transversions following DNA replication [29]. Thus, if oxidative stress is a major cause of somatic mtDNA mutations, there should be a preponderance of G∶C to T∶A transversion mutations in our samples. However, G∶C to T∶A transversions represented less than 10% of the mutations detected, regardless of the age of animals (**Figure 3**). Moreover, the frequency of G∶C to T∶A transversions did not increase appreciably with age (5% in young flies and 8% in old flies), and thus are not a major contributor to the increase in mtDNA mutation frequency with age. The most common mutation type detected in our samples was G∶C to A∶T transitions (80% of mutations in young flies and 86% in old flies). Further analysis revealed that the G∶C to A∶T transition mutations exhibited a strand bias, whereby G to A transitions occur more frequently on the major strand (the coding strand for the majority of mitochondrial genes) in both young and old tissues (**Figure S5**). Because strand asymmetric mutation accumulation has been postulated to reflect different mutation susceptibilities of the leading and lagging strands during DNA replication [33], our findings suggest that somatic mtDNA mutations occur primarily during mtDNA replication in *Drosophila*.

Biochemical studies indicate that 8-oxo-dG lesions block DNA replication, which may result in mtDNA depletion [34]. Depletion of mtDNA caused by 8-oxo-dG lesions could, therefore, possibly explain the finding that G∶C to T∶A transversion mutations did not increase appreciably with age. To address this hypothesis, we analyzed mtDNA copy number in fly heads using qPCR. We found no difference in mtDNA copy number relative to nuclear DNA between young and old flies (**Figure S6**). Therefore, mtDNA depletion does not account for the relative lack of an age-related increase in G∶C to T∶A mutation frequency.

To further explore the influence of oxidative stress on somatic mtDNA mutations in *Drosophila*, we tested whether flies deficient in the oxidative stress response pathway accumulate somatic mtDNA mutations at a higher rate. Superoxide anion is primarily detoxified in mitochondria by mitochondrially localized manganese superoxide dismutase 2 (Sod2). While null alleles of the *Drosophila Sod2* gene exist, *Sod2* null homozygotes survive for only a few hours as adults [35]. To circumvent this limitation, we generated transheterozygous flies that bear the *Sod2^n283^* null mutation in combination with a hypomorphic mutation of the *Sod2* gene (*Sod2^wk^*). Studies of *Sod2^n283^/Sod2^wk^* transheterozygotes (hereafter referred to as *Sod2* mutants) have shown that these mutants exhibit increased oxidative stress, but are viable for nearly 50 days as adults [36].

**Figure 4:**
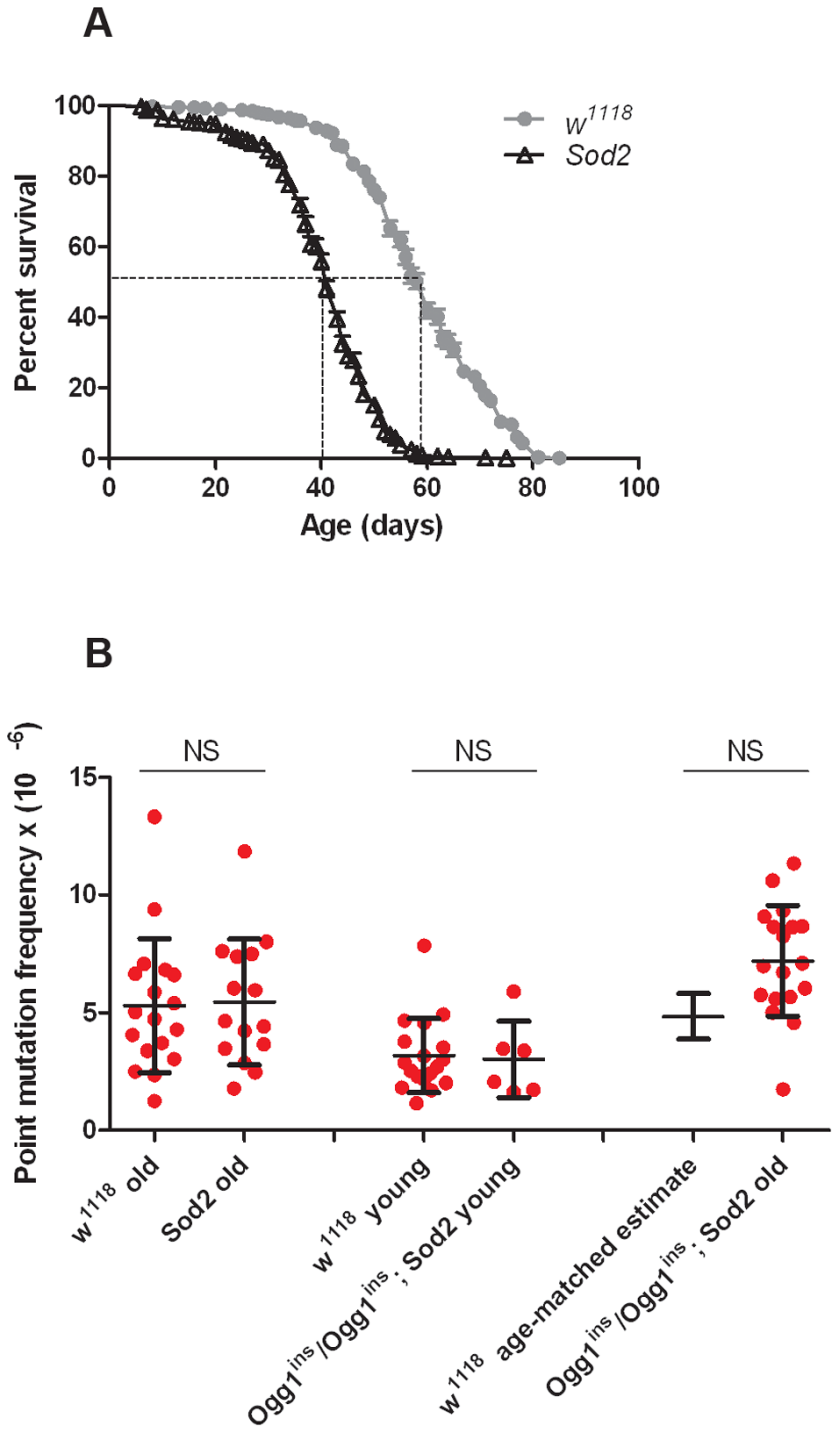
Oxidative stress response pathway mutants do not have an increased mtDNA mutation frequency. (**A**) Lifespan of *w^1118^* and *Sod2* mutant flies. The median lifespan (indicated by dashed lines) was 60 days for *w^1118^* flies and 41 days for *Sod2* mutants. (**B**) The mutation frequency using pooled data from all three *TaqI* sites in old *Sod2* mutants (39- to 41-day-old), young *Ogg1^ins^*/*Ogg1^ins^; Sod2* double mutants (1- to 3- day-old), old *Ogg1^ins^*/*Ogg1^ins^*; *Sod2* double mutants (26- to 28- day-old), and age-matched *w^1118^* controls. The 26- to 28- day-old control estimate was obtained from linear regression of the age-dependent *w^1118^* mutation frequency. Horizontal bars represent the median mutation frequency, and error bars indicate the interquartile range. There was no significant difference in mutation frequency between mutants and age-matched controls (Mann-Whitney unpaired U tests).

Before conducting RMC analysis on *Sod2* mutants, we verified that they would be suitable for our studies by performing a lifespan analysis (**Figure 4A**). We found that the median lifespan of *Sod2* mutants was 41 days and that both the median and the maximum lifespan of *Sod2* mutants were shortened by ∼30% relative to our isogenic *w^1118^* strain. We measured the somatic mtDNA mutation frequency in 40- to 42-day-old *Sod2* mutants. Because our data indicate that the somatic mtDNA mutation pattern is similar in heads and thoraces and the *Sod2* gene is ubiquitously expressed [37], we examined the mutation frequency in *Sod2* mutants using only heads. Surprisingly, we detected no increase in the mtDNA mutation frequency at any of the individual *TaqI* sites analyzed in 40- to 42-day-old *Sod2* mutants relative to age-matched control animals (**Figure S7**). Pooled data from all three *TaqI* sites also failed to reveal a difference in the mutation frequency between *Sod2* mutants and controls (**Figure 4B** **, Table S2, Table S3**). These findings provide further support for the conclusion that oxidative stress does not contribute significantly to the somatic mtDNA mutation frequency.

**Figure 5:**
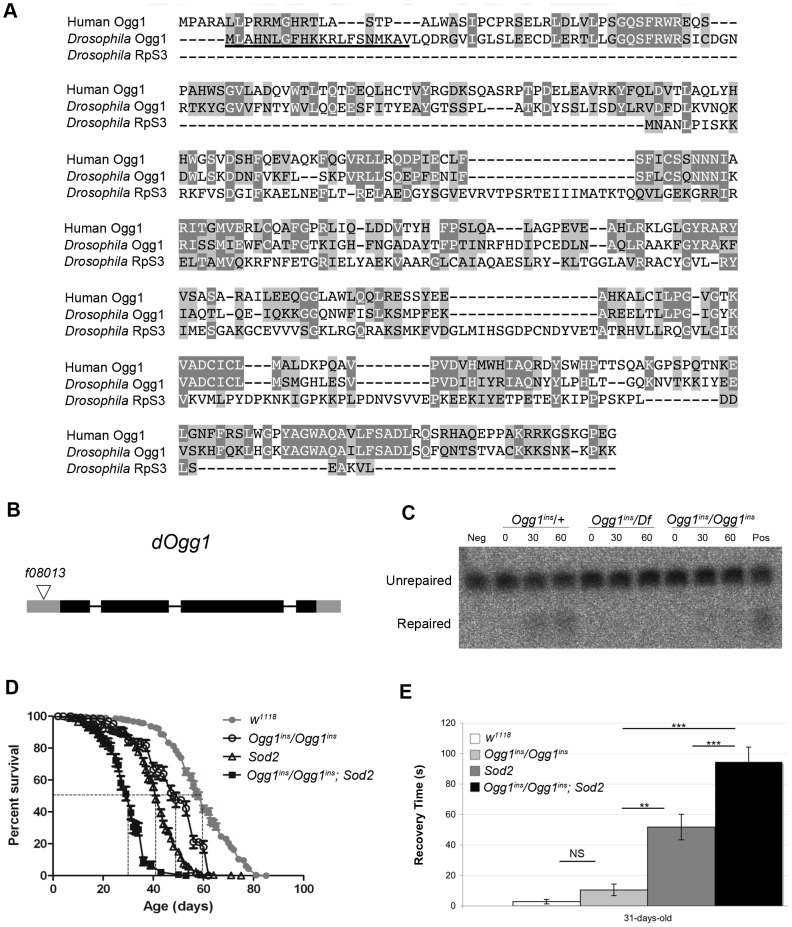
An *Ogg1* mutation diminishes Ogg1 activity and interacts genetically with *Sod2*. (**A**) Amino acid sequence alignment of human Ogg1 with *Drosophila* Ogg1 and RpS3. Dark shading denotes amino acid identities; light shading denotes amino acid similarities. The putative N-terminal mitochondrial targeting sequence of *Drosophila* Ogg1 is underlined. (**B**) The *Ogg1* gene comprises four exons, designated by rectangles, and the coding sequence is indicated with dark gray shading. The *Ogg1^f08013^ piggyBac* insertion (designated by a triangle) is located 78 base pairs upstream of the *Ogg1* translation start site. (**C**) 8-oxo-dG repair activity was assayed by monitoring cleavage of a ^32^P-labeled synthetic probe containing 8-oxo-dG, using protein extracts from flies of the indicated genotypes (Df = the *Df(1)BSC627* deletion that removes *Ogg1*; ins = the *Ogg1^f08013^ piggyBac* insertion). Results of the assay are shown following 0, 30, and 60 minutes of incubation. Neg = No protein; Pos = Mouse brain protein extract. (**D**) Lifespan of *w^1118^*, *Ogg1^ins^/Ogg1^ins^*, *Sod2*, and *Ogg1^ins^/Ogg1^ins^*; *Sod2* double mutant flies. The median lifespan (indicated by dashed lines) was 60 days for *w^1118^*, 49 days for *Ogg1^ins^* mutants, 41 days for *Sod2* mutants, and 31 days for *Ogg1^ins^*; *Sod2* double mutants. All lifespan curves are statistically different from one another using log rank tests (*p*<0.001). (**E**) *w^1118^*, *Ogg1^ins^/Ogg1^ins^*, *Sod2*, and *Ogg1^ins^/Ogg1^ins^*; *Sod2* double mutant flies were subjected to a mechanical stress sensitivity test at 31 days. Values represent mean time (seconds) of recovery from mechanical stress, and error bars represent standard error of the mean. Significance was determined using one-way ANOVA followed by Tukey's post hoc tests (**p<0.01; ***p<0.001).

A caveat of our findings with *Sod2* mutants is that efficient repair of oxidatively damaged bases might account for the lack of effect of Sod2 deficiency on the mtDNA mutation frequency. Previous work indicates that the 8-oxo-dG lesions formed by superoxide anion attack on mtDNA are primarily removed by a mitochondrially localized 8-oxoguanine glycosylase [32], [38]. Studies in *Drosophila* indicate that there are two genes, *dOgg1* (referred to as *Ogg1*) and *Ribosomal protein S3* (*RpS3*) that encode 8-oxoguanine glycosylase activity [39]. However, only the Ogg1 protein sequence bears significant homology to the human Ogg1 protein, and only Ogg1 appears to contain an N-terminal mitochondrial targeting sequence (**Figure 5A**). These findings suggest that Ogg1 is primarily responsible for the repair of mitochondrial 8-oxo-dG lesions in *Drosophila*.

To test the hypothesis that efficient repair of 8-oxo-dG lesions explains the lack of increased mutation frequency in *Sod2* mutants, we inactivated Ogg1 in an *Sod2* mutant background. A search of FlyBase revealed an *Ogg1* allele (*Ogg1^f08013^*; hereafter referred to as *Ogg1^ins^*) caused by a *piggyBac* transposable element insertion located 78 bp upstream of the translation start site in the *Ogg1* gene, as well as a large deletion (*Df(1)BSC627*) that removes the Ogg1 gene [40] (**Figure 5B**). We prepared protein lysates from *Ogg1^ins^* heterozygotes (*Ogg1^ins^*/+), *Ogg1^ins^* homozygotes (*Ogg1^ins^*/*Ogg1^ins^*), and *Ogg1^ins^* hemizygotes (*Ogg1^ins^*/*Df(1)BSC627*) and used them to assay 8-oxo-dG DNA repair activity. We found that *Ogg1^ins^* homozygotes and hemizygotes had reduced DNA repair activity relative to the heterozygous control (**Figure 5C**). The magnitude of this defect was greater in extracts from *Ogg1^ins^* hemizygotes relative to *Ogg1^ins^* homozygotes, indicating that the *Ogg1^ins^* mutation represents a hypomorphic loss-of-function allele of the *Ogg1* gene.

To test whether *Ogg1* activity masks a somatic mtDNA mutator phenotype of *Sod2* mutants, we constructed and analyzed *Ogg1^ins^/Ogg1^ins^; Sod2* double mutants. Behavioral studies of the double mutants revealed that the *Ogg1^ins^* mutation reduced lifespan and enhanced stress-induced seizure (bang-sensitive) phenotypes of *Sod2* mutants (**Figure 5D, E**), suggesting that these genes act in a common pathway. To test whether these genetic interactions reflect an increased mtDNA mutation frequency in the double mutants, we measured the mtDNA mutation frequency in the heads of young (1- to 3-day-old) and old (26- to 28-day-old) *Ogg1^ins^/Ogg1^ins^; Sod2* double mutants. We used 26- to 28-day-old animals as our “old” sample, because this is near the median lifespan of the double mutants (**Figure 5D**). The mtDNA mutation frequency at the corresponding age in *w^1118^* controls was obtained using linear regression (**Figure S3A**). To increase our ability to detect small differences, we pooled data from all three *TaqI* sites analyzed. Despite this measure, we were unable to detect a difference in somatic mtDNA mutation frequency between young double mutants and age-matched controls (**Figure 4B**). While there was a trend towards an increase in somatic mtDNA mutations in old double mutants, this difference did not reach significance (p = 0.08). Together, our findings indicate that oxidative stress is not a major contributor to the somatic mtDNA mutation frequency in *Drosophila*.

## Discussion

The accumulation of somatic mtDNA mutations is thought to contribute to aging and common age-related diseases, including cancer and Parkinson's disease. The causes of somatic mtDNA mutations are poorly understood, as are the factors that influence their cellular frequency following their occurrence. Given the powerful genetic tools available in *Drosophila* for studying fundamental biological processes, we explored the utility of this model system to investigate the mechanisms that influence the frequency of somatic mtDNA mutations. We show that features associated with mtDNA mutations in vertebrates are conserved in *Drosophila*, including the somatic mtDNA mutation frequency, an increased frequency of somatic mtDNA mutations with age, a prevalence of G∶C to A∶T transition mutations, and possibly, clonal expansion of somatic mtDNA mutations. These findings indicate that *Drosophila* will serve as a valuable model system in which to examine the mechanisms influencing somatic mtDNA mutation frequency.

Although oxidative stress is commonly cited as a major contributor to somatic mtDNA mutations [31], our work does not support this idea. The fraction of G∶C to T∶A transversion mutations, a consequence of 8-oxo-dG lesions, is small in young flies and does not increase appreciably with age. These findings parallel recent results in mice and humans showing a similarly low occurrence of G∶C to T∶A transversion mutations in samples from both young and old individuals [25], [41], [42]. Furthermore, we found that mutations in genes that oppose superoxide-induced DNA damage do not detectably influence the mtDNA point mutation frequency. However, because the total number of somatic mtDNA mutations increases with age but the percentage of G∶C to T∶A transversions remained nearly constant, our data do leave open the possibility that oxidative stress is a minor contributor to the increased burden of somatic mtDNA mutations with age. Moreover, while our studies indicate that superoxide is not a major cause of mtDNA point mutations, further work will be required to determine whether superoxide contributes to the frequency of mtDNA deletions, and to assess the influence of other forms of ROS, such as hydroxyl radicals, on mtDNA mutation frequency.

How can we reconcile our current findings with the body of work supporting a role of ROS in mtDNA mutations? Such work includes direct sequencing of mtDNA [43], [44], and a study showing that catalase expression in mouse mitochondria reduced the frequency of mtDNA mutations [45]. One possible explanation for at least some of the conflicts involving sequencing is that PCR amplification of DNA that contains 8-oxo-dG lesions results in G∶C to T∶A transversion mutations during the amplification process. Sequencing of material containing amplification-induced mutations could thus result in an overestimation of the true G∶C to T∶A transversion frequency. Further compounding this potential problem is the finding that oxidative modification of mtDNA can occur during mtDNA isolation [46]. By contrast, the RMC assay is immune to this type of artifact because *TaqI* is capable of digesting oxidized DNA, and thereby removes oxidized molecules prior to mutation detection by qPCR [18], [25]. Given that the study involving catalase ovexpression also used RMC to monitor mtDNA mutation frequency, we cannot presently offer an explanation for this conflict. However, it is important to emphasize that the findings from studies of catalase overexpression do not necessarily represent a direct conflict with our current conclusion because our findings leave open the possibility that ROS makes a small contribution to the mtDNA mutation frequency. Nevertheless, further studies will be required to explain the sizeable magnitude of the effect of catalase overexpression on the mtDNA mutation frequency, given the small contribution of ROS to mtDNA mutations suggested by our work.

If oxidative stress accounts for only a small fraction of the somatic mtDNA mutation burden, what then is the major source of these mutations? Previous work has shown that expression of a proofreading-deficient form of mtDNA polymerase (Pol-γ) causes primarily G∶C to A∶T transition mutations [47]. This finding raises the possibility that Pol-γ misincorporation errors are responsible for the G∶C to A∶T mutations that account for the majority of the somatic mtDNA mutations detected in our work. However, while Pol-γ replication errors may account for some of the G∶C to A∶T transition mutations that occur in somatic tissues, this model is difficult to reconcile with the finding that most of these mutations accumulate in a strand asymmetric fashion. Instead, we propose that most of the G∶C to A∶T transition mutations that occur in somatic tissues arise because of differing sensitivities of the leading and lagging strands to mutagenesis during mtDNA replication. In *Drosophila*, the mtDNA minor strand is replicated in a continuous fashion (the leading strand) and the major strand is replicated in a discontinuous fashion (the lagging strand) [48]–[50]. During leading strand synthesis, the template for the lagging strand (the minor strand) is transiently single-stranded, and thus more susceptible to DNA damage than the template for the leading strand (the major strand). One type of DNA damage that is compatible with our findings is spontaneous deamination of cytosine [51]–[53], the rate of which increases several orders of magnitude in single-stranded DNA [33]. Selective cytosine deamination on the minor strand during leading strand synthesis would offer an explanation for the preponderance of C to T mutations that we detect on the minor strand (equivalent to G to A mutations on the major strand). While transcription can also lead to asymmetric accumulation of mutations on the non-transcribed strand [54]–[56], the pattern of mutational accumulation observed in our work is inconsistent with a transcriptional origin because most of the C to T mutations occur on the transcribed strand. The strand asymmetric mutational bias detected in our study is consistent with previous findings in aging human brain [42] and in the *Drosophila* germline [57], suggesting that spontaneous cytosine deamination on the lagging strand template during replication is a conserved mechanism of mtDNA mutation accumulation.

Another important question that is raised by our findings is why *Ogg1* mutations enhance *Sod2* mutant phenotypes if the double mutants do not have a significantly higher mtDNA mutation frequency than control flies. While we believe that ROS-mediated damage to proteins and/or lipids [35], [36] is the primary explanation for the shortened lifespan and bang sensitivity of *Sod2* mutants, the genetic interaction between the *Sod2* and *Ogg1* mutations cannot readily be explained by an increase in protein or lipid damage in the double mutants because there is no evidence that Ogg1 is involved in the repair of damaged proteins or lipids. However, recent studies show that several other factors involved in base excision repair also function in apoptosis and transcriptional regulation [58], [59]. Thus, one possibility is that Ogg1 has a function other than DNA repair, and that the loss of this function is responsible for the genetic interaction with Sod2. Alternatively, Ogg1 may also play a role in nuclear DNA repair, and the genetic interaction between *Ogg1* and *Sod2* may reflect inefficient repair of oxidatively damaged nuclear DNA. Finally, the genetic interaction between *Ogg1* and *Sod2* may reflect an increase in the somatic mtDNA mutation frequency that was below the detection limit of our assay. Although we did not observe a statistically significant increase in the mtDNA mutation frequency in the *Ogg1; Sod2* double mutants, there was a clear trend in this direction. Future studies will be required to distinguish between these possibilities.

In conclusion, our study sheds additional light on the genesis of mtDNA mutations, and also indicates that *Drosophila* is a tractable genetic model system with which to study the mechanisms that influence the frequency of mtDNA mutations. Little is known of the cellular factors that influence heteroplasmy, clonal expansion, and the inheritance of mtDNA mutations. The use of *Drosophila* should facilitate rapid advancement in our understanding of mtDNA mutations and their roles in aging and disease.

## Materials and Methods

### Fly strains and maintenance

Flies were maintained on standard cornmeal-molasses food (70% cornmeal, 10.5% molasses, 2.5% yeast, and 1% agar) at 25°C. The *w^1118^* isogenic fly strain, *Ogg1 PiggyBac* element insertion (*Ogg1^f08013^*) strain, and *Ogg1* deletion (Df(1)BSC627) strain were obtained from the Bloomington *Drosophila* Stock Center. The *Sod2^n283^* and *Sod2^wk^* mutants have been described previously [35], [36].

### Behavioral analysis

#### Lifespan analysis

One- to two-day-old flies were placed in vials in groups of up to 20 flies. Every 2–3 days flies were transferred to fresh vials and the number of deaths was recorded. Each lifespan trial was repeated at least three times, with a minimum of 100 flies per genotype analyzed. Survivorship was plotted as a function of time (in days) using GraphPad Prism software version 5.04 (GraphPad Software, Inc., La Jolla, CA). Survival curves were compared between genotypes using the log-rank test to determine significance.

#### Bang sensitivity analysis

Flies were tested for sensitivity to mechanical stress using a modified version of a published procedure [60]. Briefly, one- to two-day-old flies were placed into vials in groups of 2 and aged to 31 days. Fly vials were inverted and vortexed on the maximum setting for ten seconds, and the time required for flies to recover from paralysis and/or seizures was recorded. A minimum of 20 flies was tested per genotype.

### 5-bromo-2′-deoxyuridine (BrdU) labeling and analysis

One-day-old flies were collected, starved for 24 hours, and then transferred to vials lined with Whatman filter paper (GE Healthcare, Pittsburg, PA). Flies were fed BrdU by adding a solution of 1 mg/mL BrdU (Sigma-Aldrich, St. Louis, MO) in 5% sucrose and 5% yeast directly to the filter paper. Fresh BrdU containing solution was added to the filter paper twice a day for three days. Following BrdU labeling, tissues were collected for Southwestern blot analysis or confocal microscopy.

#### Southwestern analysis

Flies were flash frozen in liquid nitrogen and vortexed for 10 to 15 seconds to dissociate body parts. One hundred fly heads were collected and homogenized in 300 µL buffer (10 mM Tris-HCl pH 8.0, 150 mM NaCl, 20 mM EDTA, 1% SDS) in a 1.5-mL Eppendorf tube using a plastic pestle. RNase A (Applied Biosystems, Foster City, CA) was added to a final concentration of 10 µg/mL and incubated at 37°C for 30 minutes. Following RNase A treatment, proteinase K (Qiagen, Valencia, CA) was added to a final concentration of 200 µg/mL and incubated either overnight at 37°C or for three hours at 56°C. An equal volume of cold phenol/chloroform/isoamyl alcohol (25∶24∶1; Fisher, Pittsburgh, PA) was then added, and the samples were inverted ten times, mixed, and centrifuged at 15,800× *g* for 10 minutes. The aqueous phase containing the DNA was collected and the phenol/chloroform/isoamyl alcohol extraction was repeated once, followed by a single extraction with chloroform. The DNA was then precipitated with 3M sodium acetate pH 5.2 and isopropanol and centrifuged at 15,800× *g* for 25 minutes at 4°C. The DNA pellet was resuspended in 10 mM Tris-HCl and 0.1 mM EDTA (pH 8.0), and 3 µg of DNA was immediately digested with BglII (New England Biolabs, Beverly, MA) for at least 16 hours at 37°C. The digested DNA was then subjected to Southern blot analysis using nylon membranes (Millipore Corp, Billerica, MA). DNA crosslinking to membranes was performed using a UV Stratalinker 1800 on the auto crosslink setting (Agilent Technologies, Santa Clara, CA). The membrane was blocked for 1 hour in 5% milk in PBS at 25°C, then incubated with antiserum to BrdU (Abcam #92837, Cambridge, MA) at a 1∶1000 dilution overnight. Membranes were then washed in 0.1% Tween in PBS, followed by incubation with HRP-conjugated anti-chicken secondary antibody (Sigma-Aldrich #A9046, St. Louis, MO) at a 1∶80,000 dilution. Western Blotting Substrate (Fisher, Pittsburgh, PA) was used to detect peroxidase signal.

#### Immunohistochemistry and confocal microscopy

BrdU-labeled tissues were prepared for confocal microscopy by dissecting the thoracic ganglion in cold PBS using a dissecting microscope. The thoracic ganglion was stained with 25 nM MitoTracker Deep Red for 25 minutes (Invitrogen, Carlsbad, CA), rinsed with 0.1% Triton X-100 in PBS (wash buffer), and fixed for 30 minutes in 4% paraformaldehyde (Ted Pella Inc., Redding, CA) in PBS at 4°C. After rinsing in wash buffer, the thoracic ganglion was incubated in 1 M HCl for 30 minutes at 25°C, then blocked in 0.1% Triton X-100 with 10% heat-inactivated FBS in PBS (blocking buffer) for 1 hour at 25°C. Primary antibody to BrdU (Abcam #Ab6326, Cambridge, MA) was then added at 1∶100 dilution and incubated overnight at 4°C. After washing with wash buffer, Alexa Fluor 488 goat anti-rat IgG (Molecular Probes #A11006, Eugene, OR) was added to blocking buffer at 1∶10,000 dilution and incubated for 2 hours at 25°C. Stained thoracic ganglia were washed with wash buffer, mounted in Fluoromount (Sigma-Aldrich, St. Louis, MO), and imaged using an Olympus FV-1000 with a 100× lens and 2–4× digital zoom.

### Random mutation capture assay

#### qPCR primer design and validation

*TaqI* test primers were designed to flank *TaqI* sites and amplify products sized between 95 and 250 bp using NCBI's Primer-BLAST program (Primer3, [61]) (Primer-Blast, National Center for Biotechnology Information, National Library of Medicine, Bethesda, MD). The Primer-BLAST algorithm was also used to optimize primer specificity by testing for mis-priming to related nuclear and mitochondrial sequences. There are four known *Drosophila* mitochondrial pseudogenes that reside in the nuclear DNA [62], and these sequences were excluded from primer design to ensure that our primers were specific for mitochondrial sequences. Control primers were designed in the same manner as test primers but do not flank a *TaqI* site. Because the *Drosophila* mtDNA has a high A∶T content, control primers were sought at mtDNA sequences with a relatively high G∶C content.

We validated the efficiency of control and test primer pairs by measuring the cycle threshold (Ct; the cycle number at which the signal rises above background) using a four-fold dilution series of undigested mtDNA, and plotting Ct vs. dilution (**Table S1**). Theory predicts that product abundance doubles at each cycle of PCR amplification, so the slope of Ct vs. dilution should be 2. Primer pairs that deviated by 15% or more from a slope value of 2 were discarded. Only test and control primers that yielded slope values within 5% of one another were used in our analyses (**Table S1**). Finally, we sequenced the PCR product produced by each primer pair that passed our quality control thresholds to ensure that only the correct target was amplified.

#### DNA isolation

One- to three-day-old flies were collected and transferred to fresh food every 2–3 days until they reached the ages chosen for RMC analysis. Flies were then flash frozen in liquid nitrogen in groups of 100 and vortexed for 10 to 15 seconds to dissociate body parts. Heads were collected, and the thorax was sectioned from the abdomen using a razor blade. Groups of 100 heads or thoraces were then processed to isolate DNA as described above (Southwestern analysis). The DNA pellet was resuspended in 10 mM Tris-HCl and 0.1 mM EDTA (pH 8.0), and was then used for *TaqI* digestion or frozen at −80°C until use.

TaqI digestion: The methods for the RMC assay are described in detail in reference [19]. Briefly, each sample of ∼10 µg of DNA was treated with 100 U of *TaqI* restriction enzyme (New England Biolabs, Beverly, MA) and incubated at 65°C. An additional 100 U were added every hour for a total of 10 hours to promote complete DNA digestion.

#### qPCR experiments

qPCR experiments were carried out on an MJ Research DNA Engine Opticon 2 System (Hercules, CA) using Power SYBR Green PCR Master Mix (Agilent, Wilmington, DE). The PCR cycle consisted of an initial denaturing step at 95°C for 10 minutes, followed by 45 cycles of denaturing at 95°C for 30 seconds, annealing at 60°C for 1 min, and extension at 72°C for 15 sec. After the 45 cycles of PCR, a final extension step was performed at 72°C for 1 min. Melting curves were performed on all PCR products and analyzed using the Bio-Rad Opticon Monitor II software to ensure that primers amplified a single product.

#### Mutation quantification

To quantify the mutation frequency, we first used the Bio-Rad Opticon Monitor II software to determine the Ct values of the control primers (Ct_control_), representing total mtDNA molecules, and the Ct values of the test primers (Ct_test_), representing mutant mtDNA molecules. The ratio of mutant to WT mtDNA molecules was then calculated from the difference in Ct values using the following equation: Point mutation frequency = Mutant molecules/WT molecules = 1/2^ΔCt^×4, where ΔCt = Ct_control_−Ct_test_. This value is multiplied by a factor of 4 because mutations at any of the four bases in the *TaqI* restriction site would prevent digestion. Following qPCR amplifcation, the amplification products were either sequenced or digested with *TaqI* and subjected to densitometry to estimate the percentage of contaminating WT molecules that remained. This percentage was then used to adjust the mtDNA mutation calculation to account for contaminating WT mtDNA.

### Clonal expansion

To test for clonal expansion, we cloned DNA from eight individual samples, performed Sanger sequencing on at least 10 colonies per sample, and quantified the percentage of each type of mutation detected. Data was collected from the *TaqI* sites located in the *mt:Cyt-b* or *mt:CoI* genes. Additionally, we compared the total amount of mtDNA and total number of mtDNA copies in outlier and non-outliers samples. This analysis revealed that neither of these parameters differed between outliers and non-outliers (**Table S4**).

### DNA copy number

Total DNA was isolated as described above (Random Mutation Capture Assay: DNA isolation) from the heads of young and old flies and used as template in qPCR. qPCR settings were as described above (Random Mutation Capture Assay: qPCR experiments). Relative copy number of mtDNA to nuclear DNA was determined using control mtDNA primers against *mt:CoIII* (see **Table S1**) and nuclear DNA primers against *β-Tubulin* [63].

### Ogg1 repair of 8-oxo-dG

Ogg1 activity was assayed using fly head homogenates as previously described [64]. Briefly, the heads from 15–18 anesthetized flies, aged 1 to 3 days, were collected on ice using a razor blade and homogenized in 20 mM Tris-HCl (pH 8.0), 1 mM EDTA, 1 mM DTT, 0.5 mM spermine, 0.5 mM spermidine, protease inhibitor, and 50% glycerol. A 1∶10 volume of 2.5 M KCl was added to this homogenate and mixed at 4°C for 30 minutes. Samples were then centrifuged at 15,800× *g* for 30 minutes and the supernatant was collected and frozen at −80°C until use in the 8-oxo-dG repair assay. Ogg1 activity was measured at 37°C using 30 µg of protein extract and a ^32^P-labeled synthetic probe containing 8-oxo-dG for the indicated times. The reaction was stopped by placing the solution on ice and adding 20 µl of loading buffer containing 90% formamide, 10 mM of NaOH, and Blue/Orange Loading Dye (Promega Corp., Madison, WI). The solution was then heated at 95°C for 3 min and placed on ice. The samples were loaded onto a polyacrylamide gel (20%) in 7 M urea and 1× TBE running buffer and run at 400 mV for 2 h. An FLA-3000 Series Fuji Film Fluorescent Image Analyzer was used to obtain images from the gel. All reagents used in this analysis were obtained from Sigma-Aldrich unless indicated otherwise (St. Louis, MO).

### Sequence algorithms

Clustal Omega (Cambridge, UK) was used to align human Ogg1 and *Drosophila* Ogg1 and RpS3 sequences [65], [66], and MitoPROT (Munich, Germany) was used to identify N-terminal mitochondrial targeting sequences in *Drosophila* Ogg1 [67]. Snap Gene software was used to create the *Drosophila* mtDNA map with *TaqI* sites (GSL Biotech LLC, Chicago, IL)

### Statistics

Significance was determined using Mann-Whitney unpaired U-tests to compare mtDNA mutation frequencies; log-rank test to compare survival curves for lifespan analyses; a one-way ANOVA followed by Tukey's post hoc test for bang-sensitive experiments; a two-tailed Student's t-test for mtDNA copy number comparison, and Pearson's correlation coefficient for linear correlation of mtDNA mutation frequency with age.

## Supporting Information

Figure S1 BrdU labeling of mtDNA provides support for mtDNA replication in *Drosophila* somatic tissues. (**A**) DNA was isolated from the heads of BrdU-fed flies, digested with BglII, then subjected to Southwestern blot analysis using an anti-BrdU antiserum. A single 19.5 kb band was detected in BrdU-fed flies, consistent with the size expected for linearized *Drosophila* mtDNA. (**B**) Thoracic ganglia from BrdU-fed and untreated control flies were labeled with MitoTracker Red and a primary antibody against BrdU (green) and subjected to confocal imaging. While non-specific labeling of BrdU is present in control images, colocalization (yellow) of BrdU with mitochondria (arrows) is present only in BrdU-fed flies. Scale bar = 2 µm.
(TIF)

Figure S2 Lifespan analysis and mutation frequencies of an isogenic *w^1118^* strain. (**A**) The median lifespan of *w^1118^* flies was 60 days. Horizontal bars indicate age ranges selected for RMC analysis. The mtDNA mutation frequency at the three *TaqI* sites in (**B**) heads and thoraces of young animals, (**C**) heads of young and old animals, (**D**) thoraces of young and old animals, and (**E**) heads and thoraces of old animals. Horizontal bars represent the median mutation frequency, and error bars indicate the interquartile range.
(TIF)

Figure S3 The mtDNA mutation frequency as a function of age. The point mutation frequency determined from RMC analysis is plotted relative to ages in heads (**A**) and thoraces (**B**). Mutation frequency data from heads fit best to a model of linear increase as a function of age. Data from thoraces could not be reliably fit to any specific model due to the broad fluctuation between samples. Linear correlation was determined using Pearson's correlation coefficient (r).
(TIF)

Figure S4 Clonal expansion of mtDNA mutations may explain variance in outlier samples. Histograms show the relative proportions of the mtDNA mutations detected in outlier and non-outlier samples. The mtDNA mutation frequency, age (young or old), tissue type (head or thorax), and *TaqI* sites (*mt:Cyt-b* or *mt:CoI*) are indicated. The wild-type *TaqI* sequence (TCGA) is shown for reference, and the mutations detected by sequencing are color-coded as indicated. In four out of five outlier samples, a single mutation accounts for >85% of all mutations detected, while no single mutation exceeded 80% in non-outlier samples.
(TIF)

Figure S5 *Drosophila* mtDNA exhibits mutational strand bias. Horizontal bars indicate the number of mtDNA mutations of the indicated type on the minor and major strands in young (n = 6) and old tissues (n = 7). G to A transitions are significantly more frequent on the major strand than on the minor strand in young and old tissues. None of the other mutation types exhibited significant strand asymmetry. Significance was determined using a Students t-test, and error bars represent standard error of the mean.
(TIF)

Figure S6 Mitochondrial DNA copy number does not decrease with age. Total DNA from the heads of young or old *w^1118^* flies was subjected to qPCR to determine the mtDNA copy number relative to nuclear DNA. No significant difference in the mtDNA∶nuclear DNA ratio was detected between young (n = 4) and old flies (n = 4) (p = 0.3). Significance was determined using a Student's t-test and error bars represent standard deviation.
(TIF)

Figure S7 The mutation frequency is not greater in *Sod2* mutants at separate *TaqI* sites. The mtDNA mutation frequency at the three *TaqI* sites in heads of old *Sod2* mutant and age-matched *w^1118^* animals. Horizontal bars represent the median mutation frequency, and error bars indicate the interquartile range. There was no significant difference in mutation frequency between mutants and age-matched controls (Mann-Whitney unpaired U tests).
(TIF)

Table S1 RMC test primers and usability in RMC assay. The 33 *TaqI* sites in *Drosophila* mtDNA are indicated according to their position relative to the first base pair in the mitochondrial genome and the gene in which they reside. The sequences of all primer pairs designed for use in the RMC assay are provided. Test primer pairs flanking *TaqI* sites used in the RMC assay are highlighted in bold, and coloring corresponds to that used in the legend of Figure 1. Control primer pair location and sequences are also listed.
(XLS)

Table S2 Mitochondrial DNA mutation frequencies. The mtDNA mutation frequency for each sample analyzed is listed according to genotype (*w^1118^*, *Sod2* single mutants, or *Ogg1^ins^*/*Ogg1^ins^; Sod2* double mutants), *TaqI* site (*mt:Cyt-b, mt:CoI*, or *tRNA-Arg*) analyzed, tissue source (heads or thoraces), and age (in days).
(XLS)

Table S3 Number of base pairs screened and mutations detected. The number of base pairs screened and the number of mutations detected are indicated for each genotype (*w^1118^* flies, *Sod2* single mutants, and *Ogg1^ins^/Ogg1^ins^*; *Sod2* double mutants), age (days), tissue source (heads or thoraces), number of independent biological replicates, and *TaqI* site (*mt:Cyt-b*, *mt:CoI*, or *tRNA-Arg*) analyzed.
(XLS)

Table S4 mtDNA abundance is indistinguishable in outlier and non-outliers samples. Amount of DNA isolated (ug) and total number of mtDNA molecules were determined for outlier and non-outlier samples, and no significant difference was detected. Significance was determined using a Student's t-test (n = 5 for outliers and n = 3 for non-outliers).
(XLS)
