# Supplementary figures and images for: Oxidative Stress Is Not a Major Contributor to Somatic Mitochondrial DNA Mutations
Source: PLoS Genet. 2014 Feb 6;10(2):e1003974. doi: 10.1371/journal.pgen.1003974 (PMC3916223; doi:10.1371/journal.pgen.1003974)

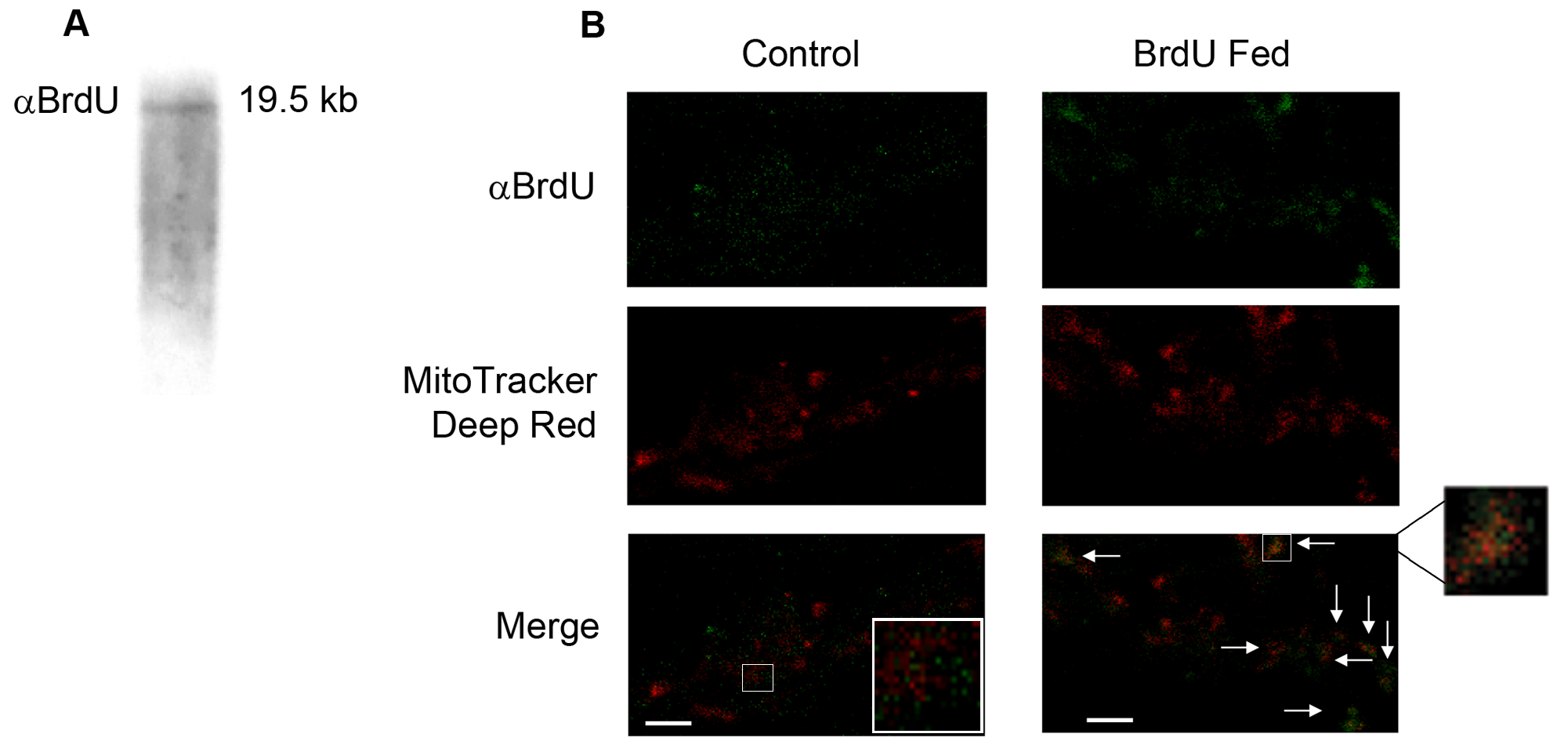

Supplement: Figure S1 — BrdU labeling of mtDNA provides support for mtDNA replication in Drosophila somatic tissues. (A) DNA was isolated from the heads of BrdU-fed flies, digested with BglII, then subjected to Southwestern blot analysis using an anti-BrdU antiserum. A single 19.5 kb band was detected in BrdU-fed flies, consistent with the size expected for linearized Drosophila mtDNA. (B) Thoracic ganglia from BrdU-fed and untreated control flies were labeled with MitoTracker Red and a primary antibody against BrdU (green) and subjected to confocal imaging. While non-specific labeling of BrdU is present in control images, colocalization (yellow) of BrdU with mitochondria (arrows) is present only in BrdU-fed flies. Scale bar = 2 µm. (TIF) [file pgen.1003974.s001.tif]

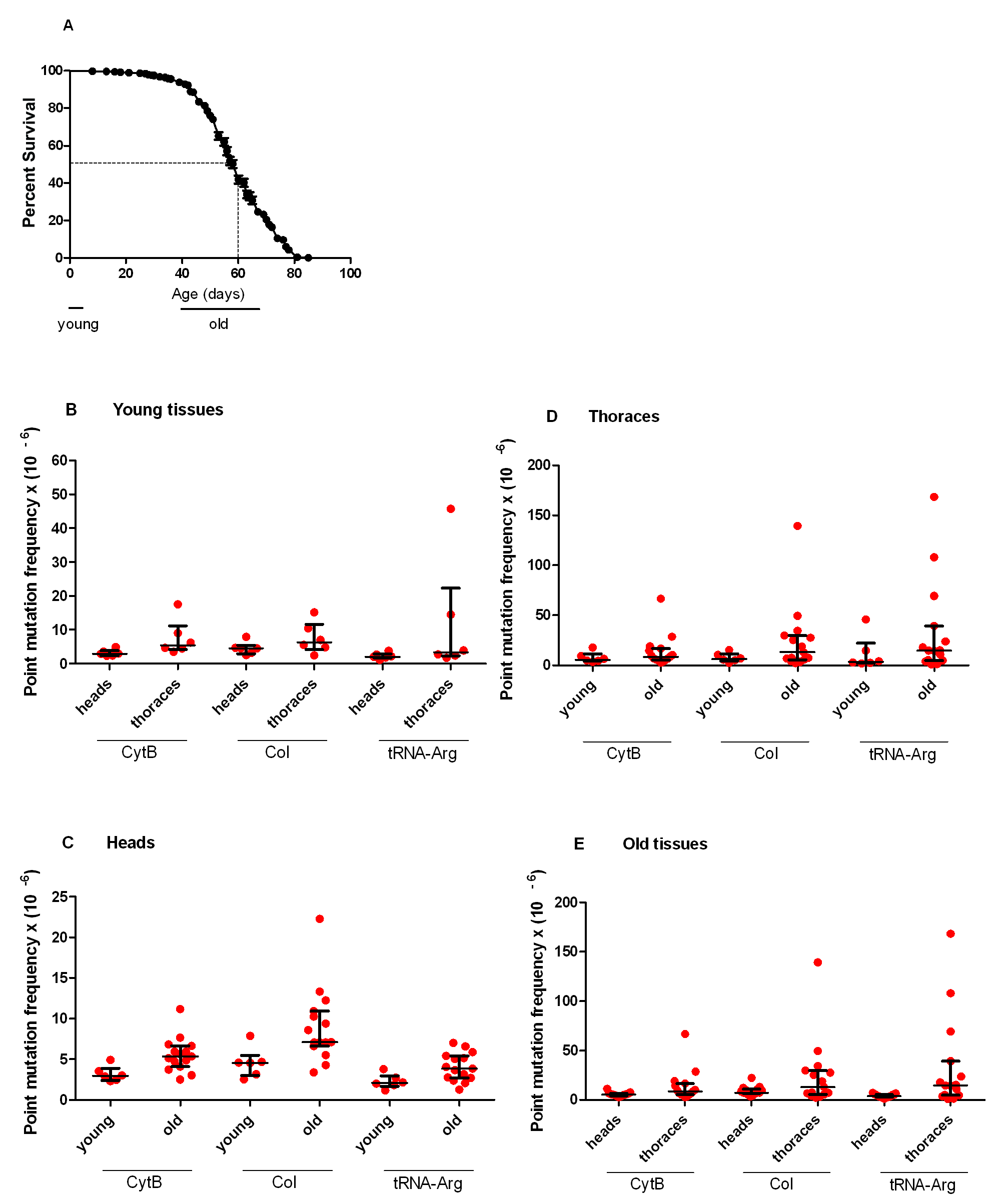

Supplement: Figure S2 — Lifespan analysis and mutation frequencies of an isogenic w1118 strain. (A) The median lifespan of w1118 flies was 60 days. Horizontal bars indicate age ranges selected for RMC analysis. The mtDNA mutation frequency at the three TaqI sites in (B) heads and thoraces of young animals, (C) heads of young and old animals, (D) thoraces of young and old animals, and (E) heads and thoraces of old animals. Horizontal bars represent the median mutation frequency, and error bars indicate the interquartile range. (TIF) [file pgen.1003974.s002.tif]

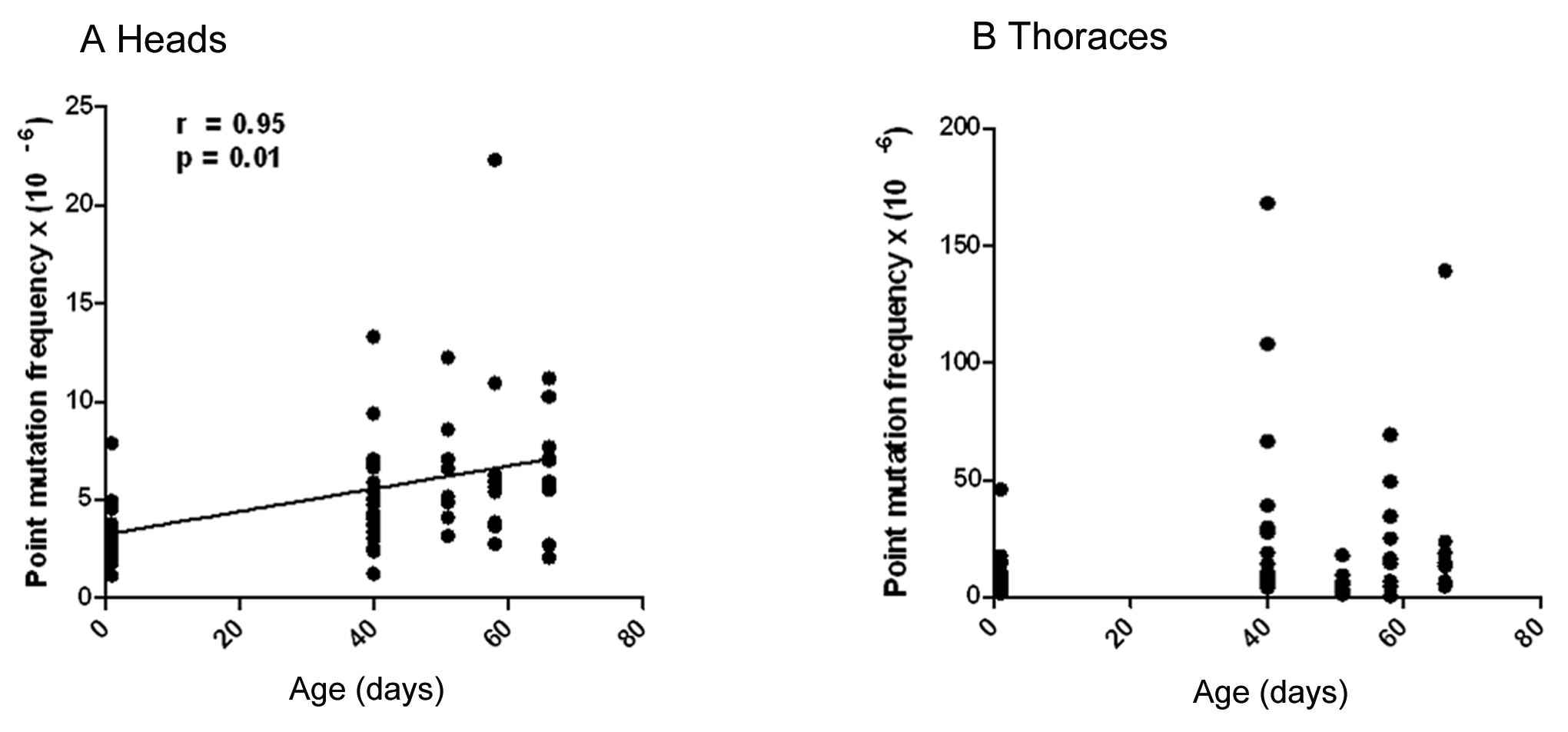

Supplement: Figure S3 — The mtDNA mutation frequency as a function of age. The point mutation frequency determined from RMC analysis is plotted relative to ages in heads (A) and thoraces (B). Mutation frequency data from heads fit best to a model of linear increase as a function of age. Data from thoraces could not be reliably fit to any specific model due to the broad fluctuation between samples. Linear correlation was determined using Pearson's correlation coefficient (r). (TIF) [file pgen.1003974.s003.tif]

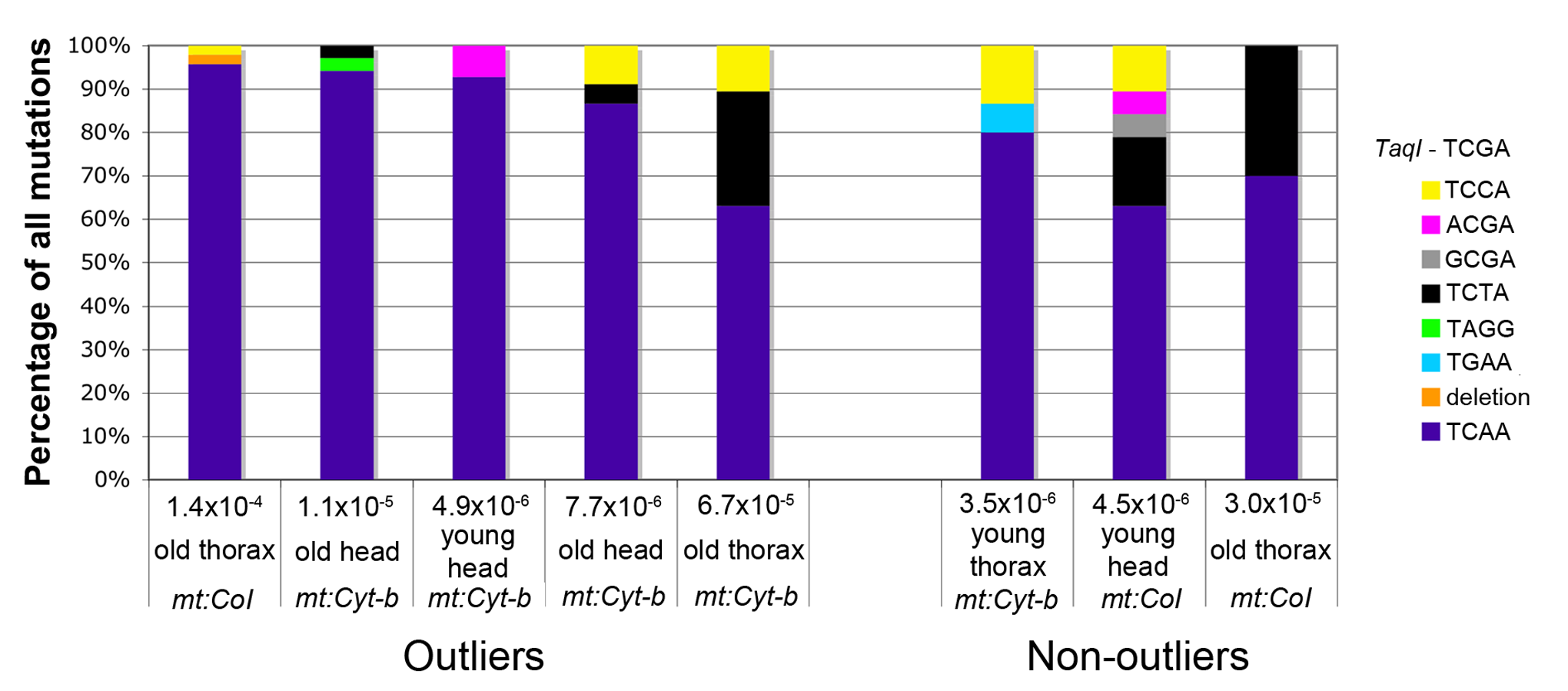

Supplement: Figure S4 — Clonal expansion of mtDNA mutations may explain variance in outlier samples. Histograms show the relative proportions of the mtDNA mutations detected in outlier and non-outlier samples. The mtDNA mutation frequency, age (young or old), tissue type (head or thorax), and TaqI sites (mt:Cyt-b or mt:CoI) are indicated. The wild-type TaqI sequence (TCGA) is shown for reference, and the mutations detected by sequencing are color-coded as indicated. In four out of five outlier samples, a single mutation accounts for >85% of all mutations detected, while no single mutation exceeded 80% in non-outlier samples. (TIF) [file pgen.1003974.s004.tif]

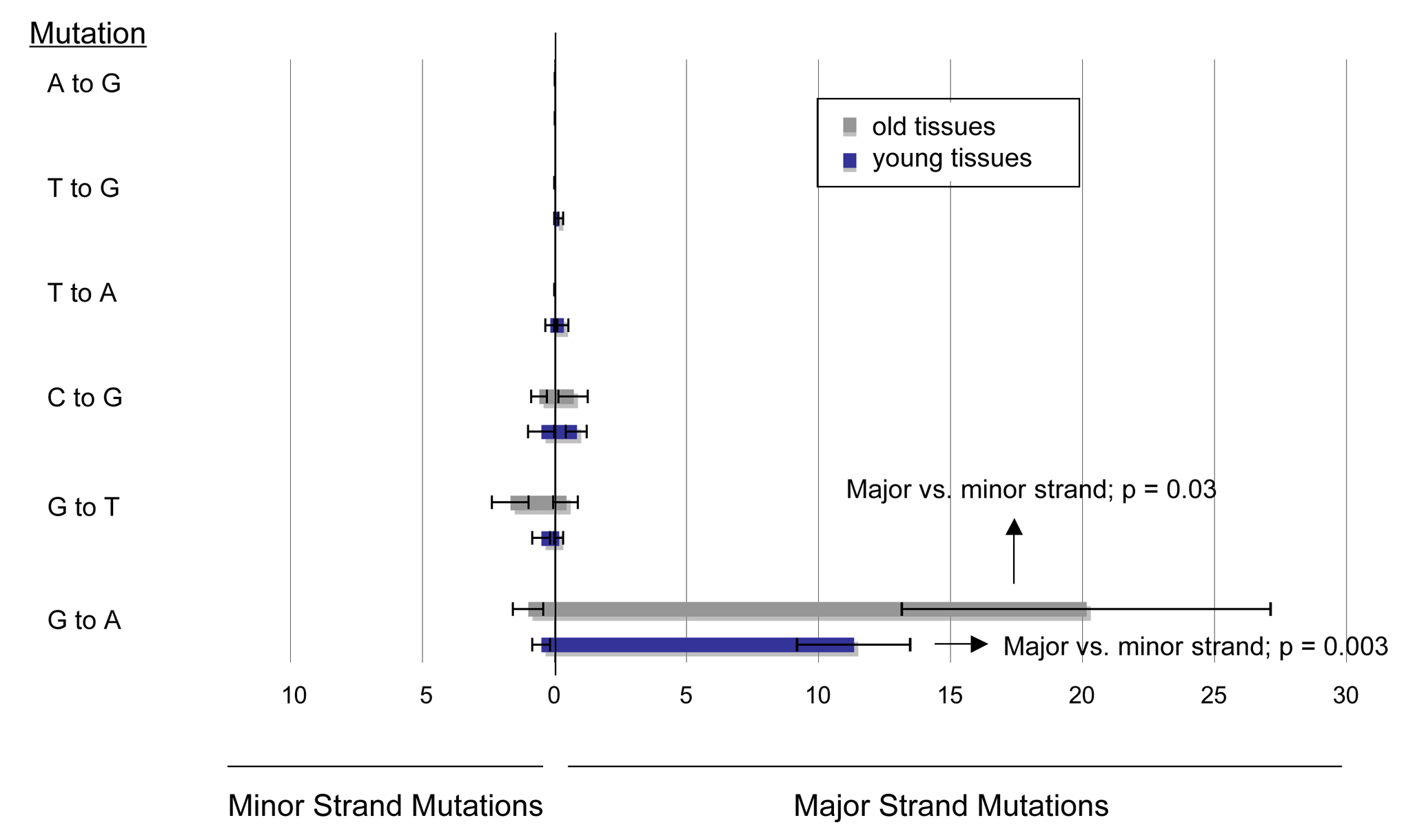

Supplement: Figure S5 — Drosophila mtDNA exhibits mutational strand bias. Horizontal bars indicate the number of mtDNA mutations of the indicated type on the minor and major strands in young (n = 6) and old tissues (n = 7). G to A transitions are significantly more frequent on the major strand than on the minor strand in young and old tissues. None of the other mutation types exhibited significant strand asymmetry. Significance was determined using a Students t-test, and error bars represent standard error of the mean. (TIF) [file pgen.1003974.s005.tif]

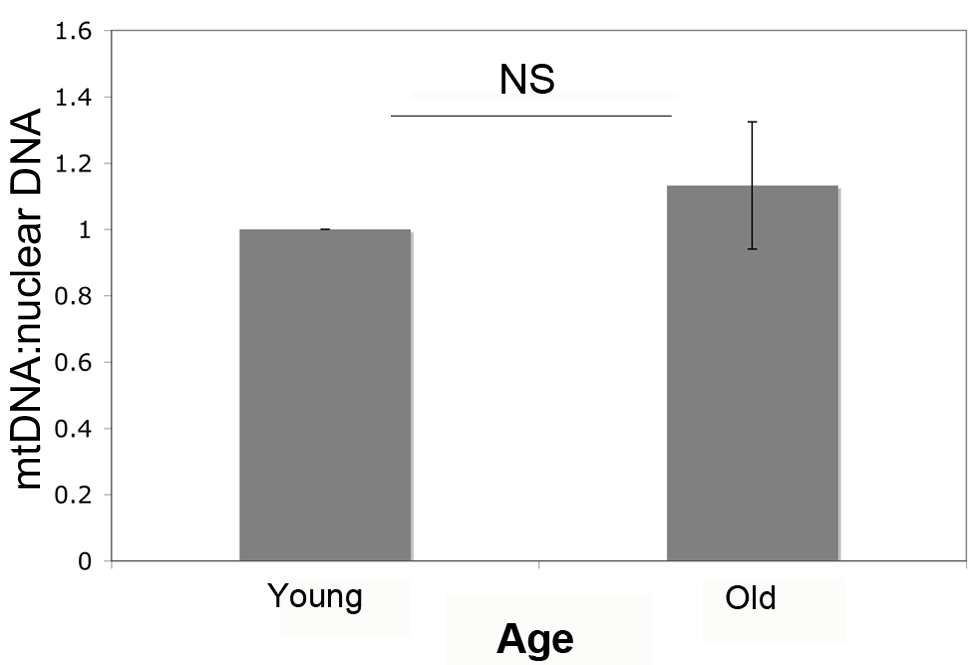

Supplement: Figure S6 — Mitochondrial DNA copy number does not decrease with age. Total DNA from the heads of young or old w1118 flies was subjected to qPCR to determine the mtDNA copy number relative to nuclear DNA. No significant difference in the mtDNA∶nuclear DNA ratio was detected between young (n = 4) and old flies (n = 4) (p = 0.3). Significance was determined using a Student's t-test and error bars represent standard deviation. (TIF) [file pgen.1003974.s006.tif]

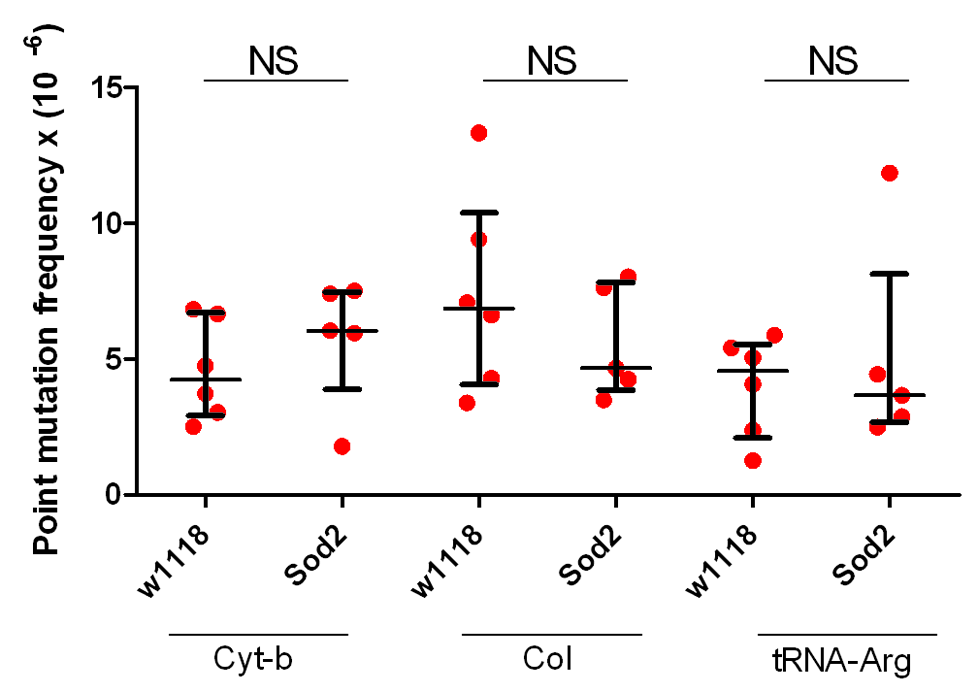

Supplement: Figure S7 — The mutation frequency is not greater in Sod2 mutants at separate TaqI sites. The mtDNA mutation frequency at the three TaqI sites in heads of old Sod2 mutant and age-matched w1118 animals. Horizontal bars represent the median mutation frequency, and error bars indicate the interquartile range. There was no significant difference in mutation frequency between mutants and age-matched controls (Mann-Whitney unpaired U tests). (TIF) [file pgen.1003974.s007.tif]
